# Preparation, Structural Characterization, and Calcium Supplementation Activity of *Lycium barbarum* Peptide–Calcium Derived from Bovine Bones

**DOI:** 10.3390/foods14223812

**Published:** 2025-11-07

**Authors:** Long Wang, Jia Cai, Lin Liu, Shunpeng Zhu, Yangxi Chen, Min Xu, Jie Zhong, Jiaxin Li, Liang Zhang, Qiang Ye

**Affiliations:** 1 Lab for Innovation & Effective Uses of Chinese Drug Germplasm Resources, Chengdu University of Traditional Chinese Medicine, Chengdu 611137, China; 18523939143@163.com (L.W.); caijia@stu.cdutem.edu.cn (J.C.); zhushunpeng@stu.cdutcm.edu.cn (S.Z.); a18081033128@163.com (Y.C.); minxu@stu.cdutcm.edu.cn (M.X.); zhongjie1@stu.cdutcm.edu.cn (J.Z.); lijiaxin1@stu.cdutcm.edu.cn (J.L.); 2 School of Pharmacy, Chengdu University of Traditional Chinese Medicine, Chengdu 611137, China; 18740825398@139.com; 3 Chengdu Institute for Drug Control, Chengdu 610000, China

**Keywords:** bovine bone, *Lycium barbarum* peptide–calcium chelate, characterization, calcium supplementation activity

## Abstract

Calcium deficiency is a global public health issue because calcium supplements are consistently inefficient. Although there is a large amount of calcium in bovine bones, its bioactivity is rather low. This study aimed to optimize the extraction process of calcium from bovine bones, develop a *Lycium barbarum* peptide–calcium chelate (LBP-Ca) preparation method, and evaluate calcium supplementation activity via pharmacodynamics tests. Response surface methodology (RSM) with a Box–Behnken design was used to screen the key parameters of the entire extraction process. The optimal conditions were determined as 1.7 mol/L citric acid, 6 h extraction time, and a material-to-liquid ratio of 1:8. The extracted calcium concentration reached 44 mg/mL. LBP-Ca was made from the extracted calcium and *Lycium barbarum* peptide. In order to confirm the formation of the chelate, it was characterized by means of UV, FT-IR, particle size, zeta potential, and SEM analysis. The results showed that the group with LBP-Ca exhibited significantly increased serum calcium levels (123.0 ± 24.5 μmol/dL) compared to the other groups in the low-calcium mice test. At the same time, LBP-Ca reduced alkaline phosphatase activity almost to normal levels and improved femur parameters and bone microstructure (higher bone volume fraction and trabecular number and better trabecular connectivity). These results indicate that LBP-Ca has superior bioavailability and bone health-promoting effects, which make it possible to develop highly effective calcium supplements from bovine bones.

## 1. Introduction

Calcium is an essential mineral nutrient for bone health, muscle function, nerve transmission, and various cellular processes for all populations across different ages and regions. Although the Earth is rather rich in calcium, calcium deficiency remains a global public health problem due to its poor absorption. According to recent global estimates, calcium inadequacy affects approximately 3.5 billion people worldwide [1,2]. In China, the 2020 National Nutrition and Chronic Disease Status Report indicated that the average calcium intake among Chinese residents is only 366 mg/day, which is significantly below the recommended 800 mg/day [3].

**Figure 1:**
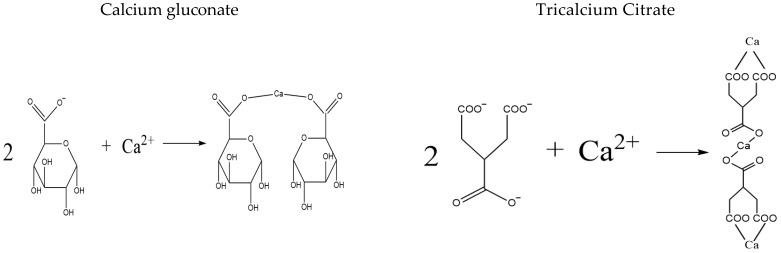
Comparison of calcium gluconate and calcium citrate.

Animal bones, particularly bovine bones, serve as an abundant source of calcium, yet the utilization rate of calcium and bioactive proteins remains low. As by-products of meat processing, bovine bones contain approximately 20–30% calcium on a dry weight basis, along with collagen—a protein that can be hydrolyzed into bioactive peptides [4,5]. Despite their significant potential, the comprehensive utilization rate of animal bones in China remains below 10% [6,7]. The efficient extraction and functional transformation of calcium from bovine bones are of paramount importance for sustainable resource utilization. Among various methods, citric acid provides exceptional advantages owing to its tricarboxylic structure, which enables it to chelate calcium ions into soluble calcium citrate, thereby preventing precipitation and enhancing process safety by minimizing equipment corrosion and preserving protein integrity. [8,9,10]. Thus, the extraction of calcium from bovine bones using citric acid represents a viable strategy for producing bioavailable calcium sources. This approach stands in contrast to conventional supplements like calcium citrate and calcium gluconate, which are often limited by poor intestinal absorption and susceptibility to dietary inhibitors, resulting in suboptimal bioavailability (Figure 1) [11,12].

Calcium supplementation has been widely adopted to address calcium deficiency. However, the bioavailability of calcium supplements varies considerably depending on their chemical forms, formulations, and administration conditions [13,14]. To address these challenges, peptide–calcium chelates have gained increasing attention in recent years. Specific amino acid sequences within peptides can coordinate calcium ions, forming stable complexes that not only enhance solubility but also facilitate absorption via intestinal peptide transporters, thereby bypassing conventional ion channels that are prone to inhibition by dietary antagonists. [15,16]. Previous studies have demonstrated that peptide–calcium chelates from various sources, including milk, soy, and marine proteins, exhibit superior calcium absorption and bone-promoting effects compared with inorganic calcium salts [17,18].

LBP-NH_2_ + LBP-COOH + Ca^2+^ ⟶ LBP-NH_2_-Ca^2+^-OOC-LBP + H^+^

**Figure 2:**
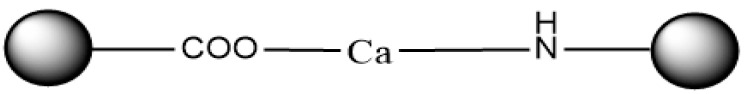
Mechanism of peptide–calcium chelation.

*Lycium barbarum*, a traditional medicinal and edible plant, has received increasing attention due to its bioactive peptides with antioxidant, immunomodulatory, and metabolic regulatory functions [19]. *Lycium barbarum* peptide (LBP) has an inherent potential to form calcium chelates. Their peptide chains carry multiple active sites capable of stably binding calcium ions via coordinate bonds [20]. Therefore, combining LBP with calcium extracted from bovine bones may not only enhance calcium absorption and utilization but also provide synergistic health benefits (Figure 2).

This study aims to establish an efficient strategy for developing functional calcium supplements with enhanced bioavailability while promoting the comprehensive utilization of bovine bone resources. To achieve this, calcium ions were extracted from bovine bones under optimized conditions using citric acid and subsequently used to prepare *Lycium barbarum* peptide–calcium chelate (LBP–Ca). The structural characteristics of LBP–Ca were characterized using multiple spectroscopic and microscopic techniques, and its calcium supplementation activity was evaluated in a low-calcium mouse model.

## 2. Materials and Methods

### 2.1. Materials

Bovine bones were provided by Sichuan Shenghe Co., Ltd. (Chengdu, China). Calcium standard solution was purchased from Sinopharm Chemical Reagent Co., Ltd. (Shanghai, China). Calcium gluconate was purchased from Hayao Group Sanjing Pharmaceutical Co., Ltd. (Harbin, China). Absolute ethanol (95% *v*/*v*), serum calcium, ALP, and protein assay kits were obtained from Solarbio Science & Technology Co., Ltd. (Beijing, China). A 0.03% low-calcium diet was purchased from Beijing Sipeifu Biotechnology Co., Ltd. (Beijing, China). *Lycium barbarum* polypeptide powder was supplied by Hongtai Biotechnology Co., Ltd. (Wuhan, China). All other chemicals and reagents were of analytical grade.

### 2.2. Screening of Acid Reagents

Hydrochloric acid, citric acid, acetic acid, lactic acid, and glycine were used to extract soluble calcium ions from bovine bones under the original extraction conditions of 4 h, 1.0 mol·L^−1^ acid concentration, and a solid-to-liquid ratio of 1:8. The influence of acid type on the yield of calcium dissolution was subsequently evaluated.

### 2.3. Optimization of Calcium Extraction by Adapting Single-Factor Experiments

Single-factor experiments were conducted to optimize key parameters: citric acid concentration (0.5, 0.75, 1.0, 1.5, 2.0 mol/L), extraction time (2, 4, 6, 8, 10, 12 h), and solid-to-liquid ratio (1:8, 1:10, 1:12).

### 2.4. Optimization of Calcium Extraction Using Response Surface Methodology

A three-factor, three-level Box–Behnken design was applied to optimize the calcium extraction process. The independent variables included citric acid concentration (X_1_: 0.5, 1.0, 1.5 mol/L), extraction time (X_2_: 2, 4, 6 h), and material-to-liquid ratio (X_3_: 1:8, 1:10, 1:12 g/mL). The response variable—calcium extraction yield (mg/mL)—was quantified using the o-cresolphthalein complexone method in accordance with the National Food Safety Standard GB 5009.92-2016 [21].

The experimental design consisted of 17 runs, including 5 replicates at the center point. The relationship between the response variable and the independent variables was modeled using a second-order polynomial equation:

(1) Y = β_0_ + Σβ_i_x_i_ + Σβ_ii_x_i_^2^ + ΣΣβ_ij_x_i_x_j_

In the model, Y represents the predicted response; β_0_ is the intercept; β_i_, the linear coefficients; β_ii_, the quadratic coefficients; and β_ij_, the interaction coefficients, with x_i_ and x_j_ denoting the coded independent variables. Design-Expert software (version 8.0.6, Stat-Ease Inc., Minneapolis, MN, USA) was used for experimental design, data analysis, and model validation. Model adequacy was assessed using the coefficient of determination (R^2^), adjusted R^2^, and the lack-of-fit test.

### 2.5. Preparation of Peptide–Calcium Chelate (LBP-Ca)

The peptide–calcium chelate was prepared following the method described by Wang et al. [22]. The hydrolysate was filtered, and the filtrate was combined with the optimized calcium extract at a protein-to-calcium ratio of 3:1. The mixture was adjusted to pH 7.0 and incubated at 37 °C for 1 h with continuous stirring. The resulting chelate was freeze-dried and stored at −20 °C until further use.

### 2.6. Structural Characterization

#### 2.6.1. UV-Vis Spectroscopy Analysis

Equal amounts of LBP and LBP-Ca were weighed and prepared into solutions with a mass concentration of 1 mg/mL. Distilled water was used as the blank control. Ultraviolet absorbance was measured using a UV-VIS spectrophotometer (Model 2550, Shimadzu, Japan) within a wavelength range of 190–500 nm, with a scanning interval of 1 nm. Spectral curves were plotted based on the recorded relationship between absorbance and wavelengths [23].

#### 2.6.2. Fourier Transform Infrared Spectroscopy (FTIR)

This experiment was conducted with slight modifications based on the method described by Beyer et al. [24]. LBP and peptide–calcium chelate were mixed with 100 mg of dry KBr, ground, and loaded onto an FTIR (Spectrum One, PerkinElmer, Waltham, MA, USA) instrument. The spectrum of each sample was scanned from 400 to 4000 cm^−1^. The obtained spectra were further processed using OMNIC 9.2 data analysis software.

#### 2.6.3. Particle Size and Zeta Potential Analysis

A dynamic light scattering (DLS) analyzer (Litesizer 500, Anton Paar, Graz, Austria) was used to measure the zeta potential and particle size distribution of LBP and LBP–Ca at room temperature. Both samples were dissolved in ultrapure water at a concentration of 1 mg/mL. Prior to measurement, the pH was adjusted with 0.1 mol/L HCl or NaOH, and the solutions were filtered through a 0.22 μm membrane. Samples were equilibrated for 30 s before analysis [25].

#### 2.6.4. Scanning Electron Microscopy Observation

An appropriate amount of polypeptide powder and freeze-dried powder of peptide–calcium chelate was evenly distributed on sample trays. After gold sputtering coating treatment, the voltage was applied to focus clearly, and images were acquired using a scanning electron microscope (Axio Imagerm2 EVO10, Zeiss, Jena, Germany) at magnifications of 8000× and 16,000× [26].

### 2.7. Animal Experiment

#### 2.7.1. Animals and Experimental Design

This study adopted the low-calcium mouse model established by Mengdi Zhao et al. [27]. Sixty 6-week-old male ICR mice (weighing 20 ± 2 g) were used in this study. The animal protocol was approved by the Sichuan Provincial Animal Care and Use Committee (License No.: SYXK (Chuan) 2025-294). After a 1-week acclimatization period, the mice were randomly assigned to 5 groups (*n* = 10 per group): a normal control group (standard diet containing 0.5% calcium), a low-calcium group (calcium-deficient diet with 0.03% calcium), and three calcium supplementation groups (calcium gluconate as the positive control, calcium citrate, and LBP–Ca). All calcium supplements (calcium gluconate, calcium citrate, and LBP–Ca) were administered via oral gavage as aqueous solutions. The dose was equivalent to the human Recommended Daily Allowance (RDA) of 800 mg Ca per 60 kg body weight, corresponding to 39.99 mg Ca/kg body weight. The normal control and low-calcium groups received an equal volume of distilled water. The intervention lasted for 4 weeks.

#### 2.7.2. Sample Collection and Analysis

At the end of the 4-week intervention, blood samples were collected from the orbital venous plexus following an overnight fast. Serum was obtained by centrifugation at 2236× *g* for 15 min and stored at −80 °C for biochemical analysis. Mice were then euthanized, and femurs were harvested, cleaned of soft tissue, and stored at −20 °C for further analysis.

Serum calcium levels were measured using the o-cresolphthalein complexone method. ALP activity was assessed using a commercial kit (Nanjing Jiancheng Bioengineering Institute, Nanjing, China), and serum protein concentration was determined by the Bradford method.

### 2.8. Femur Morphometric Analysis

The length of the left femurs was measured using a digital caliper. The femur index was calculated as the ratio of femur weight (mg) to femur length (mm).

### 2.9. Bone Densitometry Analysis

The right femurs were scanned using a micro-CT system (SkyScan 1176, Bruker, Kontich, Belgium) with the following settings: 50 kV voltage, 500 μA current, 9 μm resolution, and a rotation step of 0.7° [28]. Three-dimensional reconstruction and analysis were performed using NRecon (version 1.7.4.2) and CTAn (version 1.20.8.0) software (Bruker, Kontich, Belgium). The following parameters were quantified: bone mineral density (BMD), bone volume fraction (BV/TV), trabecular thickness (Tb.Th), trabecular number (Tb.N), and trabecular separation (Tb.Sp).

### 2.10. Statistical Analysis

Data are expressed as mean ± standard deviation (SD). Statistical analysis was conducted using SPSS 22.0 software (IBM Corp., Armonk, NY, USA). One-way analysis of variance (ANOVA) followed by Tukey’s post hoc test was used to evaluate differences among groups. Different lowercase letters (a, b, c) indicate statistically significant differences among treatment groups (*p* < 0.05).

## 3. Results

### 3.1. The Determination of Optimal Acid Reagents

**Figure 3:**
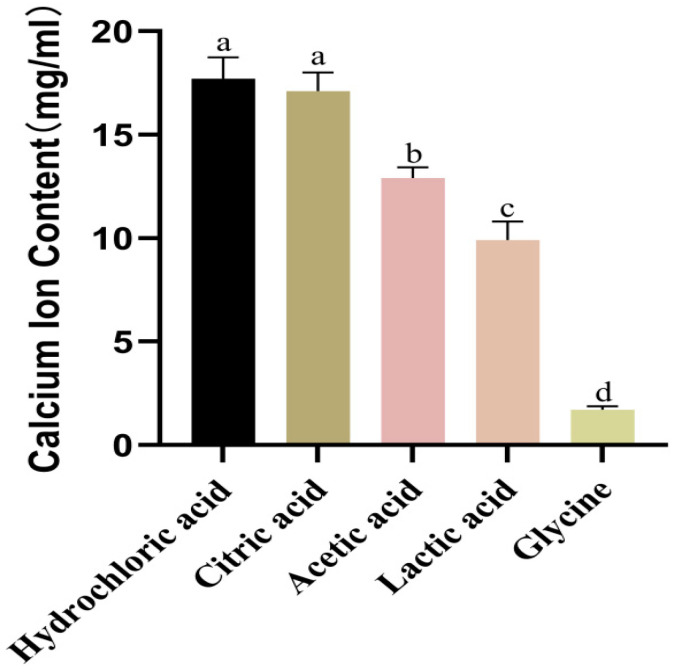
Effect of acid type on calcium ion content. Data are expressed as mean ± SD (*n* = 3). Different lowercase letters (a–d) indicate statistically significant differences (*p* < 0.05). Error bars represent standard deviation.

Figure 3 shows significant differences in Ca^2+^ release efficiency among acid treatments. Hydrochloric acid yielded the highest free calcium concentration (~18 mg/mL), but the solubilized calcium was unstable and prone to forming insoluble precipitates. In contrast, citric acid, despite a marginally lower yield, formed stable, soluble calcium–citrate complexes via multidentate chelation, which prevented precipitation. The weak dissolution capacity of acetic and lactic acids was attributed to their single dissociable proton, while glycine (near-neutral pH) was virtually ineffective. Given its superior performance in terms of calcium stability and process compatibility, citric acid was selected as the optimal reagent for calcium extraction.

### 3.2. The Optimization of Extraction Conditions of the Acid Treatment Process

**Figure 4:**
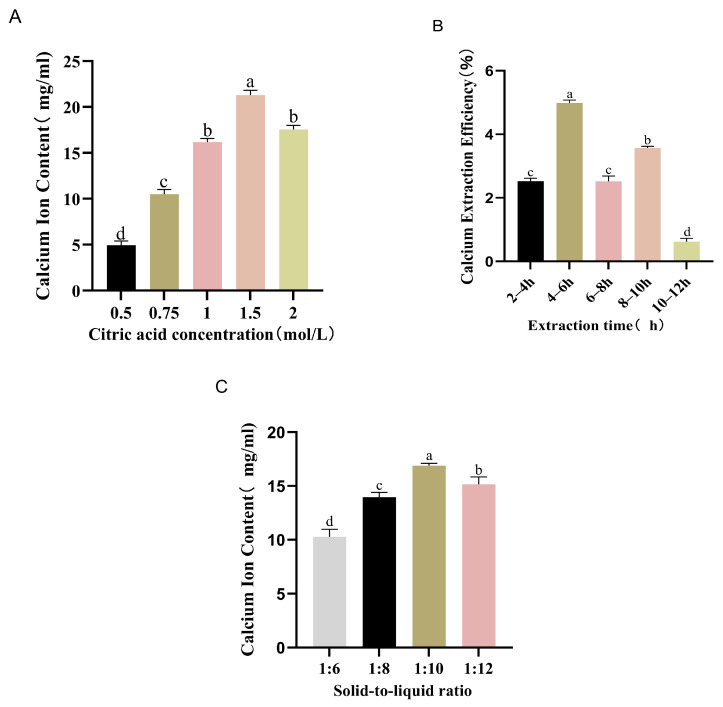
Effects of different citric acid concentrations (**A**), heating time (**B**), and solid-to-liquid ratio (**C**) on calcium ion content. All values are expressed as mean ± SD (*n* = 3). Means with the same superscript letter (a–d) are not significantly different (*p* < 0.05).

There are three key factors in the acid extraction process. To evaluate the effects of citric acid parameters on the Ca^2+^ content in bovine bone hydrolysates, single-factor experiments were conducted to examine citric acid concentration, hydrolysis time, and solid-to-liquid ratio (Figure 4A–C). The results showed that the 1.5 mol/L group yielded the highest calcium content (22 mg/mL) (Figure 4A). The fastest release rate occurred within 4–6 h among the tested time points (Figure 4B). The highest Ca^2+^ content was observed at a solid-to-liquid ratio of 1:10 (Figure 4C).

### 3.3. Optimization of Calcium Extraction Using Response Surface Methodology

The experimental design and results for the optimization of calcium extraction are presented in Table 1. The calcium extraction yield ranged from 15.2 to 44.0 mg/mL under different extraction conditions.

The second-order polynomial model describing the relationship between calcium extraction yield (Y) and the independent variables was as follows:

Y = 0.0402 + 0.0057A + 0.0044875B − 0.0014625C + 0.001725AB − 0.003675AC + 0.00375BC − 0.013525A^2^ − 0.00895B^2^ − 0.001C^2^

The ANOVA results (Table 2) indicated that the model’s *p*-value of 0.0002 (*p* < 0.01), which demonstrates the fitting equation, had excellent regression performance and high statistical significance. The linear terms A, B and the quadratic terms A^2^, B^2^ exerted a highly significant influence on the calcium content (*p* < 0.01), while the interaction terms (AC, BC) exerted a significant influence (0.01 < *p* < 0.05). Each factor influenced the results to some extent. According to the F-test, the influence of factors on calcium content followed the order A > B > C, indicating concentration > time > volume. For the regression equation, the coefficient of determination (R^2^) = 0.9677, adjusted R^2^ = 0.9261, and predicted R^2^ = 0.9196; the coefficient of variation (CV) was 9.89% (which is < 10%). These values demonstrate that the model had high reliability and could effectively reflect the actual experimental values. Adeq Precision (signal-to-noise ratio) = 13.8404, and a value > 4 is generally recognized to indicate a good fit between the model and experimental values. In conclusion, the quadratic response surface regression equation had a good fit, and the model could be used for predicting and optimizing the process conditions for calcium extraction from bovine bones.

**Figure 5:**
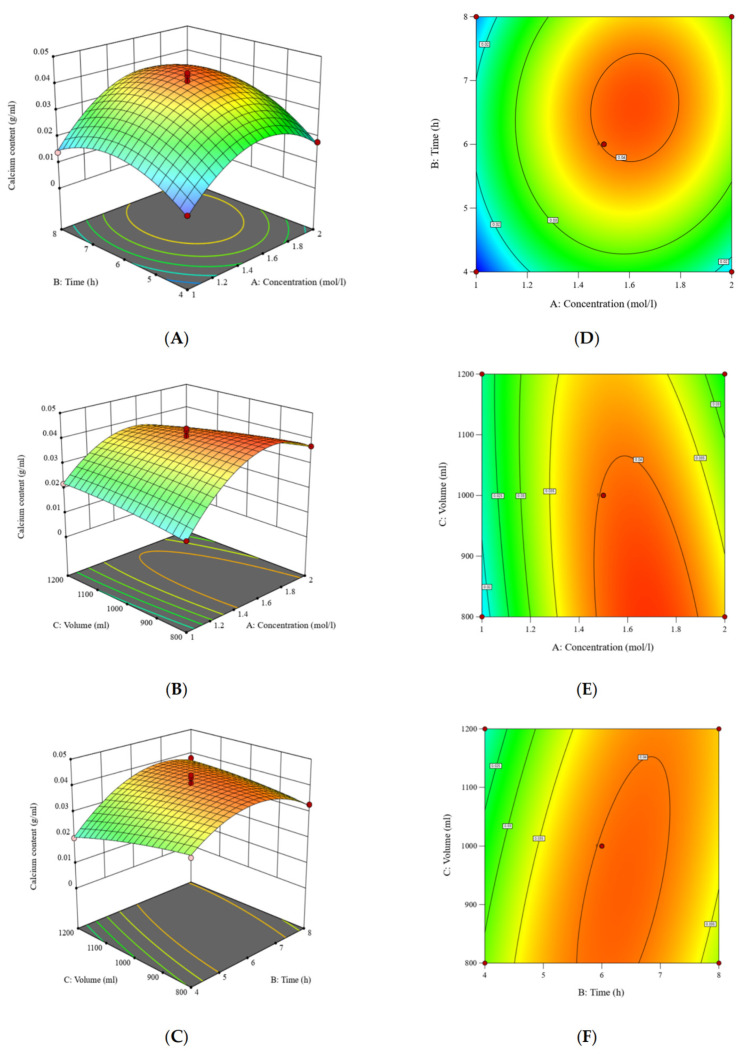
Response surface and contour plots showing the effects of the independent variables on calcium extraction yield. Surface plots for the interactions between (**A**) citric acid concentration and extraction time, (**B**) citric acid concentration and material-to-liquid ratio, and (**C**) extraction time and material-to-liquid ratio. Contour plots for the interactions between (**D**) citric acid concentration and extraction time, (**E**) citric acid concentration and material-to-liquid ratio, and (**F**) extraction time and material-to-liquid ratio.

Both the linear terms (X_1_, X_2_, X_3_) and the quadratic terms (X_1_^2^, X_2_^2^, X_3_^2^) had significant effects on calcium extraction yield (*p* < 0.05). Among the interaction terms, only the interaction between citric acid concentration and extraction time (X_1_X_2_) was significant (*p* < 0.05). The response surface plots (Figure 5) illustrate these relationships: extraction yield increased with citric acid concentration up to ~1.5 mol/L, after which it declined—likely due to calcium–citrate precipitation at higher acid levels. Similarly, yield increased with extraction time to ~6 h, beyond which no appreciable improvement was observed. The material-to-liquid ratio exerted a positive effect across the tested range, with higher ratios promoting more efficient calcium dissolution.

To ensure the reliability and authenticity of the optimization results, we adjusted the relevant parameters based on the operation of the reagents. The optimal process parameters for calcium from bovine bones are as follows: a citric acid concentration of 1.7 mol/L, an extraction time of 6 h, and a material-to-liquid ratio of 1:8, yielding a predicted extraction of 44.0 mg/mL. Validation experiments conducted under these conditions produced an actual yield of 43.8 ± 0.5 mg/mL, closely matching the prediction and confirming the model’s validity. The calcium content under different extraction conditions and in the final products is summarized in Table 3, highlighting the efficiency of the optimized acid extraction process.

### 3.4. Structural Characterization

#### 3.4.1. UV-Vis Spectroscopy Analysis

**Figure 6:**
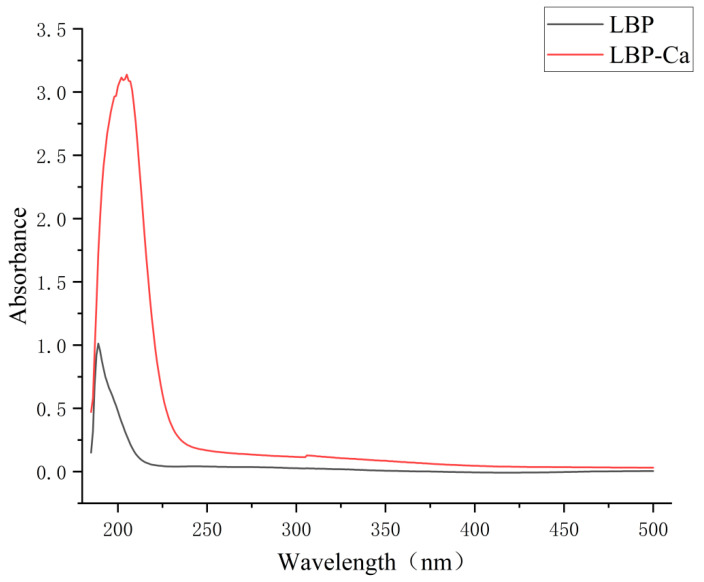
UV-Vis spectra of LBP and LBP-Ca.

UV-Vis spectroscopic intensity variations and wavelength shifts enable the assessment of specific structural features, formation of new substances, or any changes that occur [29]. As shown in Figure 6, the intense absorption band observed near 200 nm is attributed to the π→π* electronic transition of the amide chromophore, specifically the –C=O–NH– conjugated system. Compared to native LBP, the LBP–Ca complex demonstrates a markedly increased absorbance at this wavelength, a phenomenon known as the hyperchromic effect. This result is consistent with the chelation of Ca^2+^ ions at the carbonyl oxygen and amide nitrogen atoms. The coordination of Ca^2+^ enhances the polarity of the carbonyl π-electron cloud, thereby lowering the energy required for the π→π* transition and increasing the probability of such transitions. Additionally, the rigidification of the peptide backbone reduces non-radiative decay pathways, leading to an overall enhancement of the absorption signal. Moreover, the more pronounced residual absorption across 200–300 nm suggests Ca^2+^-induced extension of conjugation (greater electron delocalization) and conformational adjustments. Collectively, these spectral features indicate formation of an LBP–Ca complex with an electronic structure distinct from native LBP, which is in line with metal–peptide coordination principles.

#### 3.4.2. Fourier Transform Infrared Spectroscopy (FTIR)

**Figure 7:**
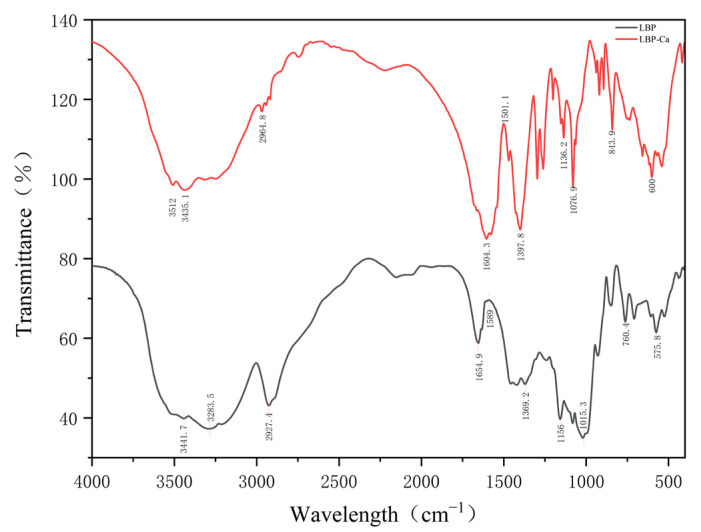
FTIR spectra.

As shown in Figure 7, the broad absorption band in LBP spanning 3500–3100 cm^−1^, centered at 3441.7 cm^−1^ with a shoulder at 3283.5 cm^−1^, is characteristic of N-H and O-H stretching vibrations, indicative of extensive hydrogen bonding. Upon chelation, this region transformed into a sharper band at 3512 cm^−1^ and a broadened band at 3435.1 cm^−1^. This shift suggests that Ca^2+^ coordination competitively disrupts the native hydrogen-bonding network, forming new N–Ca or O–Ca linkages [29]. The most critical evidence comes from the amide I region. The peak at 1654.9 cm^−1^ in LBP, assigned to C=O stretching vibrations, underwent a significant shift to 1604.3 cm^−1^ in LBP-Ca. This substantial 50 cm^−1^ redshift is a classic indicator that the carbonyl oxygen atoms are directly involved in chelating Ca^2+^ ions, as the electron density of the C=O bond is altered upon coordination [30]. Further evidence was found in the fingerprint region. The bands in LBP at 1156.0 cm^−1^ and 1015.3 cm^−1^, associated with C-O-C stretching or *p*-O vibrations (if phosphorylation is present), shifted to 1136.2 cm^−1^ and 1076.9 cm^−1^ in LBP-Ca, respectively. This implies the participation of hydroxyl, ether, or potentially phosphate groups in the chelation process [31]. Crucially, the appearance of new absorption peaks at 843.9 cm^−1^ and around 600 cm^−1^ in LBP-Ca can be assigned to Ca-O coordination stretching and metal–oxygen bending vibrations, providing definitive evidence for the formation of covalent bonds between calcium and the peptide [32].

#### 3.4.3. Particle Size and Zeta Potential

**Figure 8:**
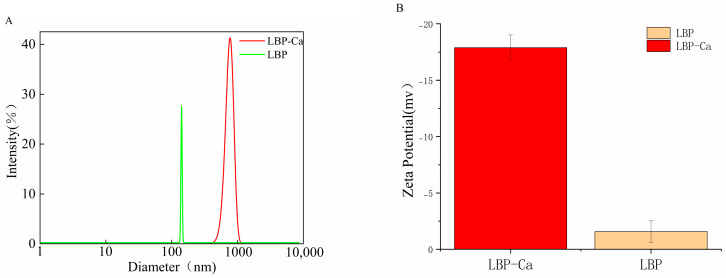
Particle size and zeta potential diagrams. (**A**) Particle size distribution of LBP and LBP–Ca; (**B**) zeta potential of LBP and LBP–Ca.

As shown in Figure 8A, the particle size distribution peak of LBP is centered at approximately 100 nm, indicating relatively small particle size and good dispersibility, whereas the distribution peak of the LBP-Ca shifts significantly to the right, centering at around 1000 nm.

Zeta potential reflects the surface charge arising from partial ionization of amino acid side chains. As shown in Figure 8B, LBP–Ca exhibited a significantly more negative ζ-potential than LBP (from −1.58 to −17.9 mV; *p* < 0.05), indicating increased net negative surface charge and stronger electrostatic repulsion. This shift is consistent with the coordination of Ca^2+^ with electron-donating groups (e.g., carbonyl and deprotonated carboxylate oxygens, and amide/hydroxyl sites), which can reorganize surface functional groups, expose acidic residues, and alter protonation equilibria [33].

#### 3.4.4. Scanning Electron Microscopy Observation

**Figure 9:**
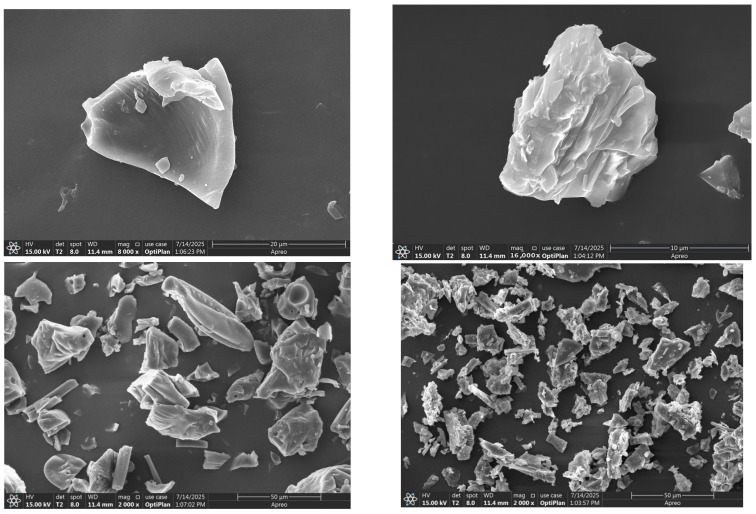
Scanning electron microscopy images.

As shown in Figure 9, LBP and LBP–Ca appear as blocky aggregates. The particles of LBP were relatively regular and flake-like, with smooth surfaces and well-defined edges, suggesting a uniform aggregation state driven by hydrogen bonding, glycosidic bonds, and hydrophobic interactions. In contrast, LBP–Ca exhibited rough surfaces with folded and laminated features, as well as irregular, fluffy morphologies, indicative of a reorganization of intermolecular interactions upon Ca^2+^ coordination. These morphological changes are likely attributed to peptide–calcium coordination bonds that disrupt the conformation and packing of LBPs [34]. Additionally, the coordination bridging between peptide amino and carboxyl groups with Ca^2+^ alters the surface properties [35]. Consistent with these observations, Hou et al. reported that in desalted duck egg white peptide–calcium systems, the presence of Ca^2+^ promotes peptide folding and aggregation, leading to the formation of stable peptide–calcium chelates [36].

### 3.5. Effects of Different Calcium Supplements on Serum Biochemical Parameters

The effects of different calcium supplements on serum biochemical parameters are presented in Table 4. The LBP-Ca group exhibited a serum calcium concentration (123.0 ± 24.5 µmol/dL) that was comparable to the positive control (calcium gluconate) group (114.5 ± 19.8 µmol/dL), with both groups showing significantly higher levels than the model and calcium citrate groups (*p* < 0.05). The low-calcium group (57.0 ± 24.1 μmol/dL) showed the lowest serum calcium level, indicating poor calcium absorption or retention.

Serum ALP, a marker of bone turnover, was markedly elevated in the low-calcium group (0.47 ± 0.06 U/mL) relative to the normal control group (0.08 ± 0.01 U/mL), indicating markedly disrupted bone metabolism under calcium deficiency. By contrast, the LBP–Ca (0.15 ± 0.02 U/mL) and calcium citrate (0.17 ± 0.03 U/mL) groups maintained ALP activities close to control levels, consistent with the stabilization of calcium homeostasis. The calcium gluconate group showed intermediate serum calcium levels (114.5 ± 19.8 μmol/dL) with ALP within the normal range, supporting its suitability for routine supplementation. Serum protein concentrations were comparable across groups (38.2–38.8 mg/mL), suggesting that calcium supplementation had no significant effect on systemic protein metabolism. The superior elevation of serum calcium by LBP–Ca likely derives from its peptide–calcium chelate architecture. Amino-acid residues coordinate with Ca^2+^ to form stable complexes that may be absorbed via intestinal peptide transport pathways (e.g., PepT1), thereby partially bypassing conventional ionic calcium uptake, which is susceptible to inhibition by dietary antagonists such as oxalates and phytates [34]. This mechanism aligns with previous studies showing enhanced calcium absorption from peptide–calcium chelates derived from various protein-derived peptide–calcium chelates [35].

### 3.6. Effects of Different Calcium Supplements on Femur Parameters

#### 3.6.1. Femur Length and Femur Index

The calcium citrate group exhibited intermediate efficacy, with a femur index (4.55 ± 0.50) significantly higher than that of the low-calcium group (*p* < 0.05) but lower than that of the calcium gluconate and LBP-Ca groups, indicating moderate bone-promoting effects (Table 5).

#### 3.6.2. Micro-CT Analysis of Bone Microstructure

**Figure 10:**
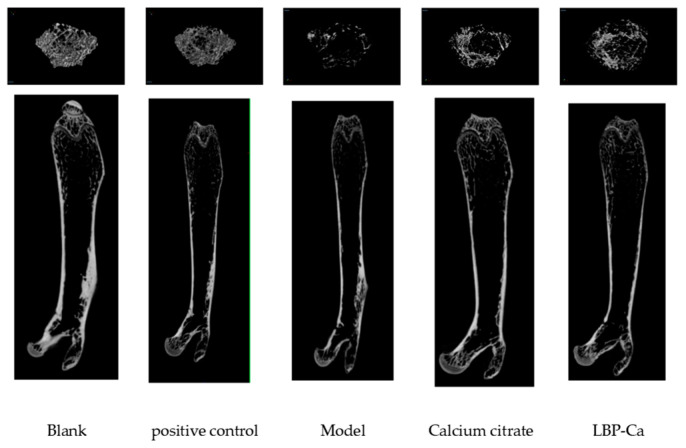
Micro-CT images of femurs from different experimental groups.

Micro-CT scanning results of the femurs from the five experimental groups are shown in Figure 10. The LBP-Ca group and the calcium citrate group maintained relatively intact trabecular structures, with dense, continuous trabecular arrangements. In contrast, the low-calcium group showed marked deterioration of the trabecular structure, characterized by numerous isolated trabeculae and structural discontinuities. Compared to the low-calcium group, the LBP-Ca group exhibited greater trabecular volume, fewer areas of trabecular deficiency, and more complete trabecular connectivity.

**Figure 11:**
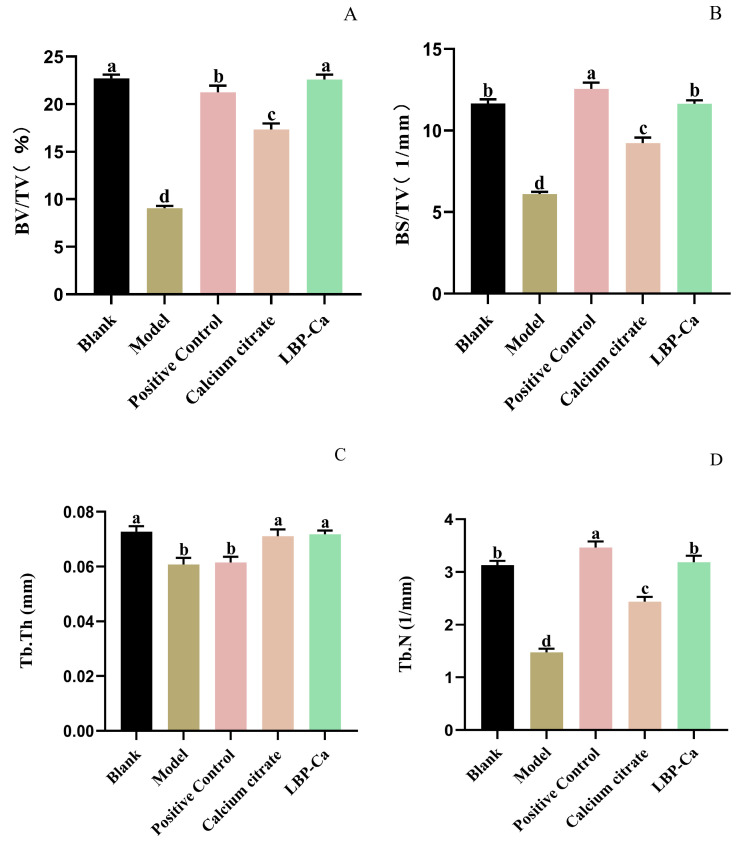

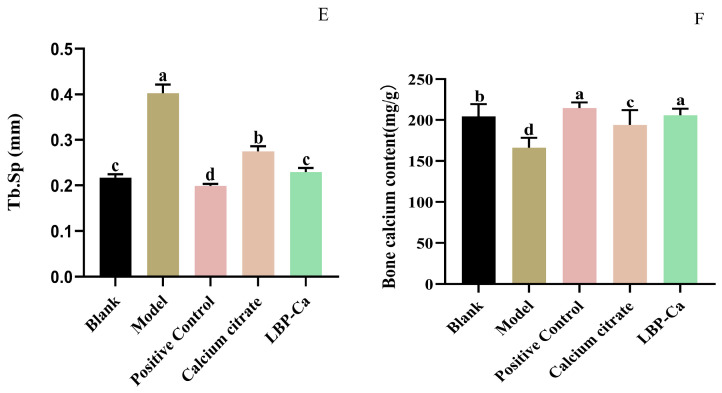
Bone morphological parameters of femurs from different experimental groups. (**A**) BV/TV; (**B**) BS/TV; (**C**) Tb.Th; (**D**) Tb.N; (**E**) Tb.Sp; (**F**) Bone calcium content. All values are presented as mean ± SD (*n* = 8).

Quantitative analysis of bone morphological parameters is presented in Figure 11. Analysis of bone microstructure confirmed the significant advantages of peptide–calcium complexes (LBP-Ca) and bovine bone-derived calcium (calcium citrate). The LBP-Ca group showed significantly higher bone volume fraction (BV/TV 22.59%) compared with the calcium citrate group and the low-calcium group. With respect to trabecular morphological parameters, trabecular thickness in the LBP-Ca group also showed favorable characteristics, exhibiting a slight advantage over the calcium citrate group. Notably, the LBP-Ca group demonstrated outstanding performance in terms of trabecular number and trabecular separation, and these parameter values were comparable to those of the calcium gluconate group. In contrast, the calcium citrate group exhibited relatively more stable regulatory effects on bone morphological parameters.

## 4. Discussion

This study successfully optimized the process for extracting calcium from bovine bones using citric acid. Although hydrochloric acid yielded the highest concentration of free Ca^2+^ due to its strong protonation capacity, the resulting free calcium ions exhibited poor stability. These ions were prone to reacting with other components to form insoluble precipitates, which led to a decrease in bioavailable calcium [36]. The strong acidity of hydrochloric acid caused severe corrosion of extraction equipment, rendering it unsuitable for use in the production of food supplements [37]. Monoprotic weak acids such as acetic acid and lactic acid could only donate one dissociable proton, leading to lower extraction ability [38]. The concentration of calcium extracted via these two acids was only 12 mg/mL and 8 mg/mL, respectively. Glycine, with a pH close to neutral, had almost no calcium dissolution capacity. Therefore, all these acids were excluded except citric acid. As a triprotic weak acid, although its calcium dissolution capacity was slightly lower than that of hydrochloric acid, its tricarboxylic acid structure could form stable and soluble calcium–citrate complexes with Ca^2+^, completely avoiding the formation of precipitates. Additionally, its moderate acidity minimizes equipment corrosion and better preserves the integrity of collagen. The extraction solution could be directly used for chelation without impurity removal. Moreover, its mild pH did not damage the structure of LBP; subsequent chelation only required adjusting the pH to 7.0 to efficiently synthesize LBP-Ca. As a widely used acidity regulator in the food industry, citric acid is among the few food additives designated as “Generally Recognized as Safe” (GRAS) [39]. It also offers advantages such as low equipment corrosion and simple residue management, thereby fulfilling multiple objectives and proving to be an ideal solvent for LBP-Ca preparation [40]. Single-factor screening and response surface methodology (RSM) were used to systematically optimize the calcium extraction process from bovine bones. The optimal parameters were determined as follows: citric acid concentration of 1.7 mol/L, extraction time of 6 h, and material-to-liquid ratio of 1:8. Under these conditions, the calcium extraction concentration was determined to be 44 mg/mL. This represents a significant improvement over traditional water extraction methods, with the calcium ion content being four times that of commercial calcium gluconate (Table 3). This method can be effectively applied in the production of calcium supplement products. Notably, this process uses bovine bones—a by-product of meat processing—thereby improving resource utilization. This is particularly important in China, where the utilization rate of animal bones is less than 10%. Additionally, the residual bone can be further used as a source of calcium for supplements, enabling high-value, comprehensive utilization of bovine bone resources.

The structural characterization using UV-Vis and FTIR spectroscopies conclusively confirms the formation of a covalent LBP-Ca chelate. The hyperchromic effect in the UV spectrum indicates Ca^2+^-induced electronic polarization of the amide chromophore [41,42]. The FTIR results provide detailed insights into the specific binding sites: the significant shift in the amide I band confirms the primary involvement of carbonyl oxygen atoms [43], while alterations in the N-H/O-H and C-O/P-O regions suggest additional coordination via amino, hydroxyl, and possibly phosphate groups [44]. The emergence of new Ca-O vibration bands acts as the definitive signature of successful chelate formation, confirming that LBP does not merely mix with calcium but forms a stable coordinated complex [45]. The reconfigured molecular structure underpins its enhanced stability and bioavailability.

Dynamic light scattering (DLS) analysis showed that LBP formed small aggregates of about 100 nm with a uniform distribution, while LBP–Ca exhibited a significant size increase to around 1000 nm. This change was attributed to Ca^2+^ acting as a bridging ion, coordinating with carboxyl, hydroxyl, and amino groups in LBP and promoting intermolecular aggregation to form larger particles, which is consistent with the reported findings in other peptide–calcium systems [46,47]. The significant shift in zeta potential from −1.58 mV to −17.9 mV indicates a significant increase in net negative surface charge, likely due to the exposure of deprotonated carboxyl groups and the formation of coordination bonds, which enhance colloidal stability and reduce the propensity for aggregation [48].

SEM offered direct visual evidence of the morphological transformations induced by chelation. LBP presented relatively smooth, flake-like particles with well-defined edges, reflecting a uniform aggregation state driven by hydrogen bonding and hydrophobic interactions. LBP-Ca exhibited a highly roughened surface with folded, laminated, and fluffy structures. This marked change in morphology could be attributed to the reorganization of intermolecular interactions upon Ca^2+^ coordination. The peptide chains, upon binding with calcium ions, likely undergo conformational changes and cross-linking, leading to the formation of larger, more porous aggregates [49,50].

In the pharmacodynamic experiments, calcium gluconate was selected as the positive control due to its extensive clinical application as an oral calcium supplement. The LBP-Ca group achieved the highest serum calcium level (123.0 ± 24.5 μmol/dL), significantly exceeding the calcium gluconate (114.5 ± 19.8 μmol/dL) and the calcium citrate (105.8 ± 22.3 μmol/dL) groups. This advantage stemmed from distinct absorption mechanisms: calcium gluconate and citrate are absorbed as free Ca^2+^ via saturable intestinal ion channels (e.g., TRPV6), whereas LBP-Ca was absorbed as an intact peptide–calcium complex through the high-capacity peptide transporter PepT1. This mechanism bypasses competition with other divalent minerals (e.g., Mg^2+^ or Fe^2+^), thereby enhancing calcium absorption [12,51]. Notably, LBP-Ca also had stronger bone-protective effects in the low-calcium mice. The low-calcium model group exhibited elevated ALP, a marker of abnormal bone turnover (characterized by increased osteoclast activity). LBP-Ca reduced ALP to 0.15 ± 0.02 U/mL—closer to the normal control group (0.08 ± 0.01 U/mL) than that of the calcium gluconate group (0.21 ± 0.04 U/mL). Micro-CT analysis further confirmed enhancements in bone microstructure, reflected by increased bone volume fraction (BV/TV, 22.59%), higher trabecular number (Tb.N, 3.15/mm), and reduced trabecular separation (Tb.Sp, 0.232 mm). These findings indicate that LBP-Ca not only facilitates calcium absorption but also modulates bone metabolism—likely attributable to the inherent bioactivity of LBP, such as activation of the Wnt/β-catenin pathway promoting osteoblast differentiation, representing a distinct advantage over conventional calcium salts [52,53].

## 5. Conclusions

This study successfully established an efficient and sustainable process for extracting calcium from bovine bones using citric acid, achieving a high calcium concentration of 44 mg/mL under the optimized conditions (1.7 mol/L citric acid, 6 h extraction, 1:8 solid-to-liquid ratio). The extracted calcium was effectively chelated with LBP to form a novel LBP–Ca complex, as confirmed by UV, FTIR, particle size, zeta potential, and SEM analyses.

Pharmacodynamic evaluations demonstrated that LBP–Ca significantly enhanced serum calcium levels, improved bone microstructure, and regulated bone metabolism markers more effectively than traditional calcium supplements. These advantages were attributed to the unique peptide-mediated absorption pathway and the inherent bioactivity of LBP.

In summary, this study provides a feasible strategy for the high-value utilization of bovine bone by-products and the development of a promising functional calcium supplement with enhanced bioavailability and bone-protective efficacy. The LBP–Ca chelate has great potential for application in functional foods and nutraceuticals aimed at addressing calcium deficiency and improving bone health.

## Figures and Tables

**Table 1:** Box–Behnken design and results for calcium extraction optimization.

Run	Citric Acid Concentration (mol/L)	Extraction Time (h)	Material-to-Liquid Ratio	Calcium Extraction Yield (g/mL)
1	1	4	1:10	0.010
2	2	4	1:10	0.018
3	1	8	1:10	0.014
4	2	8	1:10	0.030
5	1	6	1:8	0.018
6	2	6	1:8	0.037
7	1	6	1:12	0.022
8	2	6	1:12	0.026
9	1.5	4	1:8	0.030
10	1.5	8	1:8	0.033
11	1.5	4	1:12	0.020
12	1.5	8	1:12	0.038
13	1.5	6	1:10	0.041
14	1.5	6	1:10	0.035
15	1.5	6	1:10	0.043
16	1.5	6	1:10	0.038
17	1.5	6	1:10	0.044

**Table 2:** ANOVA for the response surface quadratic model.

Source	Sum of Squares	df	Meansquare	F-Value	*p*-Value
Model	0.0018	9	0.0002	23.28	0.0002
X_1_	0.0003	1	0.0003	33.01	0.0007
X_2_	0.0002	1	0.0002	20.46	0.0027
X_3_	0.0000	1	0.0000	2.15	0.1858
X_1_X_2_	0.0000	1	0.0000	1.91	0.2091
X_1_X_3_	0.0001	1	0.0001	6.73	0.0358
X_2_X_3_	0.0001	1	0.0001	6.73	0.0358
X_1_^2^	0.0008	1	0.0008	89.72	<0.0001
X_2_^2^	0.0003	1	0.0003	39.43	0.0004
X_3_^2^	5.095 × 10^−6^	1	5.095 × 10^−6^	0.6091	0.4607
Residual	0.0001	7	8.364 × 10^−6^		
Lack of Fit	3.750 × 10^−6^	3	1.250 × 10^−6^	0.0912	0.9610
Pure Error	0.0001	4	0.0000		
Cor Total	0.0018	16			
R^2^ = 0.9677 R^2^_Adj_ = 0.9261 R^2^_pre_ = 0.9196 Adeq Precision = 13.8404 C.V.% = 9.89%					

**Table 3:** Calcium content of water-extracted bovine bones, acid-extracted bovine bones, LBP-Ca, and calcium gluconate.

	Water-Extracted Bovine Bones	Acid-Extracted Bovine Bones	LBP-Ca	Calcium Gluconate
Calcium content (mg/mL)	2	44	20	11

**Table 4:** Effects of different calcium supplements on serum biochemical parameters in mice.

Group	Serum Calcium (μmol/dL)	ALP Activity (U/mL)	Serum Protein (mg/mL)
Blank	98.2 ± 18.7 ^b^	0.08 ± 0.01 ^a^	38.5 ± 0.3 ^a^
Model	57.0 ± 24.1 ^c^	0.47 ± 0.06 ^c^	38.2 ± 0.5 ^a^
Positive Control	114.5 ± 19.8 ^a^	0.21 ± 0.04 ^ab^	38.6 ± 0.4 ^a^
Calcium citrate	105.8 ± 22.3 ^b^	0.17 ± 0.03 ^ab^	38.4 ± 0.3 ^a^
LBP-Ca	123.0 ± 24.5 ^a^	0.15 ± 0.02 ^ab^	38.3 ± 0.4 ^a^

Note: Different lowercase letters (^a^, ^b^, ^c^) in the same column indicate significant differences between groups (*p* < 0.05).

**Table 5:** Effects of different calcium supplements on femur parameters in mice.

Group	Femur Length (mm)	Femur Index
Blank	16.72 ± 0.45 ^b^	4.30 ± 0.50 ^b^
Model	15.43 ± 0.32 ^a^	4.06 ± 0.50 ^c^
Positive Control	16.46 ± 0.30 ^b^	4.56 ± 0.60 ^a^
Calcium citrate	16.05 ± 0.25 ^ab^	4.55 ± 0.50 ^a^
LBP-Ca	16.68 ± 0.50 ^b^	4.11 ± 0.60 ^c^

Note: Different lowercase letters (^a–c^) in the same column indicate significant differences between groups (*p* < 0.05).

## Data Availability

The original contributions presented in this study are included in the article. Further inquiries can be directed to the corresponding author.

## References

[1] Zhang M., Liu K. (2022). Calcium Supplements and Structure–Activity Relationship of Peptide-Calcium Chelates: A Review. Food Sci. Biotechnol..

[2] Passarelli S., Free C.M., Shepon A., Beal T., Batis C., Golden C.D. (2024). Global Estimation of Dietary Micronutrient Inadequacies: A Modelling Analysis. Lancet Glob. Health.

[3] Huang L., Wang Z., Wang H., Zhao L., Jiang H., Zhang B., Ding G. (2021). Nutrition Transition and Related Health Challenges over Decades in China. Eur. J. Clin. Nutr..

[4] Shen X., Zhang M., Bhandari B., Gao Z. (2019). Novel Technologies in Utilization of Byproducts of Animal Food Processing: A Review. Crit. Rev. Food Sci. Nutr..

[5] Mezenova N.Y., Agafonova S.V., Mezenova O.Y., Baidalinova L.S., Grimm T. (2021). Obtaining and Estimating the Potential of Protein Nutraceuticals from Highly Mineralized Collagen-Containing Beef Raw Materials. Teopuя u npaкmuкa nepepaбomкu мяca.

[6] Limeneh D.Y., Tesfaye T., Ayele M., Husien N.M., Ferede E., Haile A., Mengie W., Abuhay A., Gelebo G.G., Gibril M. (2022). A Comprehensive Review on Utilization of Slaughterhouse by-Product: Current Status and Prospect. Sustainability.

[7] Wang M., Liu Y., Yao Y., Han L., Liu X. (2020). Comparative Evaluation of Bone Chars Derived from Bovine Parts: Physicochemical Properties and Copper Sorption Behavior. Sci. Total Environ..

[8] Lambros M., Tran T., Fei Q., Nicolaou M. (2022). Citric Acid: A Multifunctional Pharmaceutical Excipient. Pharmaceutics.

[9] Woodall C.M., Gomes K.V., Voigt A., Sundmacher K., Wilcox J. (2024). Tuning Acid Extraction of Magnesium and Calcium from Platinum Group Metal Tailings for Co 2 Conversion and Storage. RSC Sustain..

[10] Burgess K.A., Winpenny R.E., Saiani A., Miller A.F., Herrick A.L., Watson R.E. (2024). Citric Acid is More Effective Than Sodium Thiosulfate in Chelating Calcium in a Dissolution Model of Calcinosis. Sci. Rep..

[11] Hansen C., Werner E., Erbes H.-J., Larrat V., Kaltwasser J. (1996). Intestinal Calcium Absorption from Different Calcium Preparations: Influence of Anion and Solubility. Osteoporos. Int..

[12] Liu J., Li Y., Tang N. (2024). Enhanced Solubility and Calcium Absorption of Calcium Gluconate and Calcium Citrate in the Presence of Aspartic Acid and Glutamic Acid. J. Mol. Liq..

[13] Vavrusova M., Skibsted L.H. (2014). Calcium Nutrition. Bioavailability and Fortification. LWT-Food Sci. Technol..

[14] Straub D.A. (2007). Calcium Supplementation in Clinical Practice: A Review of Forms, Doses, and Indications. Nutr. Clin. Pract..

[15] Zhang H., Qi L., Wang X., Guo Y., Liu J., Xu Y., Liu C., Zhang C., Richel A. (2023). Preparation of a Cattle Bone Collagen Peptide–Calcium Chelate by the Ultrasound Method and Its Structural Characterization, Stability Analysis, and Bioactivity on Mc3t3-E1 Cells. Food Funct..

[16] An J., Zhang Y., Ying Z., Li H., Liu W., Wang J., Liu X. (2022). The Formation, Structural Characteristics, Absorption Pathways and Bioavailability of Calcium–Peptide Chelates. Foods.

[17] Hua P., Xiong Y., Yu Z., Liu B., Zhao L. (2019). Effect of Chlorella Pyrenoidosa Protein Hydrolysate-Calcium Chelate on Calcium Absorption Metabolism and Gut Microbiota Composition in Low-Calcium Diet-Fed Rats. Mar. Drugs.

[18] Gan J., Xiao Z., Wang K., Kong X., Du M., Wang Z., Xu B., Cheng Y. (2023). Isolation, Characterization, and Molecular Docking Analyses of Novel Calcium-Chelating Peptide from Soy Yogurt and the Study of Its Calcium Chelation Mechanism. J. Sci. Food Agric..

[19] Zhang T., Alexa E.-A., Liu G., Berisha A., Walsh R., Kelleher R. (2025). Lycium Barbarum for Health and Longevity: A Review of Its Biological Significance. Obesities.

[20] Peng J., Jia W., Zhu J. (2025). Advanced Functional Materials as Reliable Tools for Capturing Food-Derived Peptides to Optimize the Peptidomics Pre-Treatment Enrichment Workflow. Compr. Rev. Food Sci. Food Saf..

[21] (2016). National Food Safety Standard for the Determination of Calcium in Foods.

[22] Wang Y., Chen M., Liu Y., Hu Y., You J., Hu X. (2025). Characterization of Collagen Peptide-Calcium Chelate and Its Promotion of Calcium Absorption in Vitro and in Vivo. Food Biosci..

[23] Cai X., Yang Q., Lin J., Fu N., Wang S. (2017). A Specific Peptide with Calcium-Binding Capacity from Defatted *Schizochytrium* sp. Protein Hydrolysates and the Molecular Properties. Molecules.

[24] Wang D., Sun Z., Sun J., Liu F., Du L., Wang D. (2021). Preparation and Characterization of Polylactic Acid Nanofiber Films Loading Perilla Essential Oil for Antibacterial Packaging of Chilled Chicken. Int. J. Biol. Macromol..

[25] Shao J., Wang M., Zhang G., Zhang B., Hao Z. (2022). Preparation and Characterization of Sesame Peptide-Calcium Chelate with Different Molecular Weight. Int. J. Food Prop..

[26] Sun N., Wang Y., Bao Z., Cui P., Wang S., Lin S. (2020). Calcium Binding to Herring Egg Phosphopeptides: Binding Characteristics, Conformational Structure and Intermolecular Forces. Food Chem..

[27] Zhao M., Ahn D.U., Li S., Liu W., Yi S., Huang X. (2022). Effects of Phosvitin Phosphopeptide-Ca Complex Prepared by Efficient Enzymatic Hydrolysis on Calcium Absorption and Bone Deposition of Mice. Food Sci. Hum. Wellness.

[28] Sun L., Liu J., Pei H., Shi M., Chen W., Zong Y., Zhao Y., Li J., Du R., He Z. (2024). Structural Characterisation of Deer Sinew Peptides as Calcium Carriers, Their Promotion of Mc3t3-E1 Cell Proliferation and Their Effect on Bone Deposition in Mice. Food Funct..

[29] Zhao L., Huang Q., Huang S., Lin J., Wang S., Huang Y., Hong J., Rao P. (2014). Novel Peptide with a Specific Calcium-Binding Capacity from Whey Protein Hydrolysate and the Possible Chelating Mode. J. Agric. Food Chem..

[30] Dunbar R.C. (2015). Spectroscopy of Metal-Ion Complexes with Peptide-Related Ligands. Gas-Phase IR Spectrosc. Struct. Biol. Mol..

[31] Mirza Alizadeh A., Hosseini H., Mohseni M., Eskandari S., Sohrabvandi S., Hosseini M.J., Tajabadi-Ebrahimi M., Mohammadi-Kamrood M., Nahavandi S. (2021). Analytic and Chemometric Assessments of the Native Probiotic Bacteria and Inulin Effects on Bioremediation of Lead Salts. J. Sci. Food Agric..

[32] Minisha S., Johnson J., Mohammad Wabaidur S., Gupta J.K., Aftab S., Siddiqui M.R., Lai W.-C. (2023). Synthesis and Characterizations of Fe-Doped Nio Nanoparticles and Their Potential Photocatalytic Dye Degradation Activities. Sustainability.

[33] Sun N., Jin Z., Li D., Yin H., Lin S. (2017). An Exploration of the Calcium-Binding Mode of Egg White Peptide, Asp-His-Thr-Lys-Glu, and in Vitro Calcium Absorption Studies of Peptide–Calcium Complex. J. Agric. Food Chem..

[34] Wang Y., Wang R., Bai H., Wang S., Liu T., Zhang X., Wang Z. (2024). Casein Phosphopeptide Calcium Chelation: Preparation Optimization, in Vitro Gastrointestinal Simulated Digestion, and Peptide Fragment Exploration. J. Sci. Food Agric..

[35] Lin S., Li J., Hu X., Chen S., Huang H., Wu Y., Li Z. (2024). Potential Dietary Calcium Supplement: Calcium-Chelating Peptides and Peptide-Calcium Complexes Derived from Blue Food Proteins. Trends Food Sci. Technol..

[36] Shkembi B., Huppertz T. (2021). Calcium Absorption from Food Products: Food Matrix Effects. Nutrients.

[37] Aslam R., Wang Q., Yan Z. (2025). Corrosion in the Food and Beverage Industry. Industrial Corrosion: Fundamentals, Failure, Analysis and Prevention.

[38] Anyasi T., Jideani A., Edokpayi J., Anokwuru C. (2017). Application of Organic Acids in Food Preservation. Organic Acids, Characteristics, Properties and Synthesis.

[39] EFSA Panel on Additives and Products or Substances used in Animal Feed (2015). Scientific Opinion on the Safety and Efficacy of Citric Acid When Used as a Technological Additive (Preservative) for All Animal Species. EFSA J..

[40] Książek E. (2023). Citric Acid: Properties, Microbial Production, and Applications in Industries. Molecules.

[41] Narimani L., Lee V.S., Alias Y., Manan N.S., Woi P.M. (2022). Rational Design of a Fluorescent Chromophore as a Calcium Receptor Via DFT and Multivariate Approaches. Molecules.

[42] Armas A., Sonois V., Mothes E., Mazarguil H., Faller P. (2006). Zinc(II) Binds to the Neuroprotective Peptide Humanin. J. Inorg. Biochem..

[43] Kimura Y., Ono T.-A. (2001). Chelator-Induced Disappearance of Carboxylate Stretching Vibrational Modes in S_2_/S_1_ FTIR Spectrum in Oxygen-Evolving Complex of Photosystem II. Biochemistry.

[44] Berzina-Cimdina L., Borodajenko N. (2012). Research of Calcium Phosphates Using Fourier Transform Infrared Spectroscopy. Infrared Spectrosc.-Mater. Sci. Eng. Technol..

[45] Taguchi Y., Noguchi T. (2007). Drastic Changes in the Ligand Structure of the Oxygen-Evolving Mn Cluster Upon Ca^2+^ Depletion as Revealed by FTIR Difference Spectroscopy. Biochim. Biophys. Acta-Bioenerg..

[46] Kong X., Xiao Z., Chen Y., Du M., Zhang Z., Wang Z., Xu B., Cheng Y., Yu T., Gan J. (2023). Calcium-Binding Properties, Stability, and Osteogenic Ability of Phosphorylated Soy Peptide-Calcium Chelate. Front. Nutr..

[47] Sarkar P. (2017). Preparation and Characterization of Caseinophosphopeptides Mineral Complexes.

[48] Hu G., Wang D., Sun L., Su R., Corazzin M., Sun X., Dou L., Zhang M., Zhao L., Su L. (2022). Isolation, Purification and Structure Identification of a Calcium-Binding Peptide from Sheep Bone Protein Hydrolysate. Foods.

[49] Yuan F., Fu Y., Ma L., Zhu H., Yu Y., Feng X., Sun Y., Dai H., Liu X., Liu Z. (2024). Calcium-Chelating Peptides from Rabbit Bone Collagen: Characterization, Identification and Mechanism Elucidation. Food Sci. Hum. Wellness.

[50] Xu Y., Amakye W.K., Xiao G., Liu X., Ren J., Wang M. (2023). Intestinal Absorptivity-Increasing Effects of Sodium N-[8-(2-Hydroxybenzoyl) Amino]-Caprylate on Food-Derived Bioactive Peptide. Food Chem..

[51] Kellett G.L. (2011). Alternative Perspective on Intestinal Calcium Absorption: Proposed Complementary Actions of Cav1. 3 and Trpv6. Nutr. Rev..

[52] Huang W., Lao L., Deng Y., Li Z., Liao W., Duan S., Xiao S., Cao Y., Miao J. (2022). Preparation, Characterization, and Osteogenic Activity Mechanism of Casein Phosphopeptide-Calcium Chelate. Front. Nutr..

[53] Sun L., Du R., Liu J., He Z., Pei H. (2025). Emerging Therapies for Osteoporosis: A Narrative Review of Multifaceted Interventions Involving Plant-and Animal-Derived Bioactive Peptides. Aging Adv..

